# STARDUST: A pipeline for the unbiased analysis of astrocyte regional calcium dynamics

**DOI:** 10.1016/j.xpro.2024.103305

**Published:** 2024-09-12

**Authors:** Yifan Wu, Yanchao Dai, Katheryn B. Lefton, Timothy E. Holy, Thomas Papouin

**Affiliations:** 1 Department of Neuroscience, Washington University School of Medicine, St. Louis, MO 63110, USA

**Keywords:** Cell Biology, Signal Transduction, Neuroscience

## Abstract

Calcium imaging has become a popular way to probe astrocyte activity, but few techniques holistically capture discrete calcium signals occurring across the astrocyte domain. Here, we introduce STARDUST, a pipeline for the spatio-temporal analysis of regional dynamics and unbiased sorting of transients from fluorescence recordings of astrocytes. We describe steps for installing software, detecting active pixel patches, obtaining region of activity (ROA) maps, and extracting time series from ROAs. We then detail procedures for extracting signal features using custom-made code.

## Highlights

• STARDUST is a pipeline for the unbiased analysis of astrocyte calcium activity
• Steps for capturing regional calcium activity in cellular micro-domains (ROAs)
• Guidance on performing signal detection and features extraction in all ROAs
• Instructions on accessing the spatial and temporal properties of ROAs

## Abstract (editor highlights)

Publisher’s note: Undertaking any experimental protocol requires adherence to local institutional guidelines for laboratory safety and ethics.

## Graphical abstract

**Figure:**
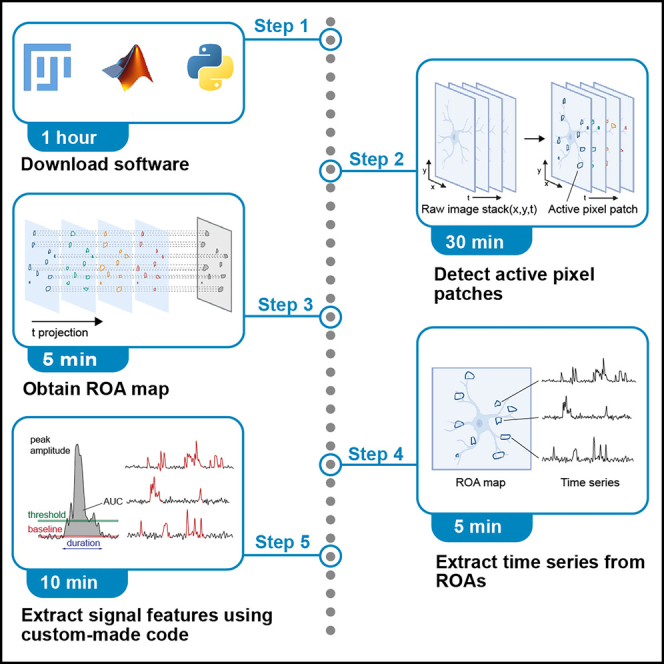

## Before you begin

Astrocytes, a main type of non-neuronal cells, display fluctuations of intracellular calcium concentrations ([Ca^2+^]_i_) that are responsive to ambient conditions and neuroactive molecules.^1^^,^^2^^,^^3^ Fluorescence imaging of Ca^2+^ indicators is thus a popular means to probe astrocyte activity *in vitro*, *ex vivo* and *in vivo*.^1^^,^^2^^,^^3^ Proven methods of neuronal Ca^2+^ imaging and analysis have been widely adapted to astrocytes for this purpose. However, important differences between neurons and astrocytes mean that key operational assumptions are not transferable. For instance, in neurons, Ca^2+^ activity is driven by depolarization-induced transmembrane Ca^2+^ fluxes. By contrast, the origins and mechanisms of astrocytes Ca^2+^ signals are diverse and still largely elusive, limiting our ability to functionally interpret Ca^2+^ events. Astrocytic [Ca^2+^]_i_ fluctuations also primarily occur outside of the soma, within highly localized micro/nano-domains, and seldom propagate or merge at the cell body.^4^^,^^5^^,^^6^^,^^7^ Concomitantly, the complexity of astrocyte morphology and its sub-diffraction limit scale also make it difficult to register areas of Ca^2+^ activity to identifiable morphological features in live fluorescence imaging. Hence, there is a growing need to enrich the astrocyte Ca^2+^ toolbox with analysis methods tailored to astrocytes. Owing to the constrains listed above, such methods need to be 1) holistic, to capture all activities in the cell, 2) unbiased, rather than restricted to user-defined regions of interest, 3) agnostic, to make no or minimal assumptions regarding the rules of Ca^2+^ propagation or integration, and 4) with instrument-limited accuracy, to favor detailed investigations consistent with the nanoscale nature of astrocytes architecture.^8^

Here, we introduce STARDUST, a pipeline that captures Ca^2+^ dynamics in confined, local micro-domains across the territory of all astrocytes in 2-photon imaging recordings. STARDUST builds upon AQuA, a popular open-source platform,^9^ to yield maps of regions of activity (ROAs) from patches of active pixels, which can be combined with cell-segmentation and/or correlated to cellular morphology. Importantly, STARDUST makes no assumptions regarding Ca^2+^ propagation across ROAs, in line with the seemingly static nature of astrocyte Ca^2+^ activity.^4^^,^^5^^,^^6^^,^^7^ Instead, STARDUST treats ROAs as independent units and focuses on decomposing Ca^2+^ dynamics in a regionalized fashion, yielding as many as 30 ROAs per cell, or thousands across a 400 × 400 μm^2^ field of view, and extracting fluorescence time-series, signals and signal features from each of them. A particular instantiation of the usefulness of STARDUST is in pharmacology experiments, where it can distinguish “stable ROAs” (active throughout the recording), from “ON ROAs” (inactive at baseline epoch and turned on during drug application), and “OFF ROAs” (active at baseline and turned off during drug application). Together, this makes STARDUST a user-friendly complement or alternative to the small number of publicly available algorithms and tools recently developed to tackle astrocyte Ca^2+^ activity.^4^^,^^6^^,^^9^^,^^10^^,^^11^ This protocol includes step-by-step guidelines, tips on how to use STARDUST and outlines multiple output examples, providing a systematic walkthrough of its core functionalities, limitation, and tunability.

### Hardware

A Microsoft Windows machine with 32 GB RAM is recommended since AQuA (steps in part 2) does not currently support Mac OS and Linux systems.

### Download AQuA

AQuA is a software package originally designed to decompose raw dynamic astrocyte imaging data into a set of Ca^2+^ ‘events’^9^ based on spatio-temporal properties of voxels and assumptions that are not necessary in STARDUST. Here, AQuA is only used to extract pixels with above-threshold fluorescence, independent of any user-defined region of interest or underlying cell morphology.

1. Download AQuA from https://github.com/yu-lab-vt/AquA. Follow the instructions in the section “Download and installation” to download the “MATLAB GUI” version to a local directory.
2. Extract all files from the zip folder “AQuA-master”.

***Note:*** To learn more about AQuA, a step-by-step user guide is available in the section “Getting started” on the AQuA GitHub page.

### Install MATLAB to run AQuA

3. Download MATLAB from https://www.mathworks.com/products/matlab.html.

**CRITICAL:** A license is required for MATLAB installation and usage. MATLAB 2017a or later is required to run AQuA.

4. Install MATLAB.

**CRITICAL:** During installation, install the following three toolboxes required for AQuA: curve fitting toolbox, image processing toolbox, and statistics and machine learning toolbox. Alternatively, install these toolboxes in “Add-Ons” in the “HOME” tab inside MATLAB.

### Download ImageJ/Fiji for image processing

5. Download ImageJ/Fiji from https://fiji.sc/.

### Install anaconda

The STARDUST code for signal detection in Part 6 is written in Python. There are several ways to run the STARDUST code. Here we provide instructions using Jupyter Notebook through Anaconda navigator. Anaconda is a Python environment that consists of an interpreter and several installed packages. Jupyter Notebook is a web application which facilitates creating and sharing documents containing live code.

6. Download Anaconda from https://www.anaconda.com/download. When clicking the download button, choose the installer version that matches the operating system.
7. Launch the installer and follow the instructions to install Anaconda.

***Note:*** The current version of Anaconda (2.6.2) contains Python 3.11 and a fully loaded Jupyter Notebook. No additional installation for Python, Python modules or Jupyter Notebook is needed. We provide a YAML file to set up a designated environment for STARDUST. For more details, please see note in step 26.

### Data collection

One channel image stack in tiff format is supported by AQuA. If the raw image file is in a different format, open the raw image file in ImageJ and convert it to tiff by *Save as > Tiff file* in ImageJ.

### Institutional permissions

All experiments were conducted in accordance with the guideline of the Institutional Animal Use Committee of Washington University in St. Louis School of Medicine (IACUC #20180184 and 21–0372, Animal Welfare Assurance #D16-00245).

## Key resources table

**Table:** 

REAGENT or RESOURCE	SOURCE	IDENTIFIER
**Deposited data**		

STARDUST example	-	https://zenodo.org/doi/10.5281/zenodo.13126733

**Software and algorithms**		

Python3, at least v3.11.0	Python Software Foundation	https://www.python.org/
Anaconda, at least v2.6.0	Anaconda, Inc.	https://www.anaconda.com/
Jupyter Notebook, v7.0.8	Kluyver et al.^12^	https://jupyter.org/
Visual Studio Code, v1.88	Microsoft	https://code.visualstudio.com/
SciPy, at least v1.11.0	Virtanen et al.^13^	https://scipy.org/
NumPy, v1.26.4	Harris et al.^14^	https://numpy.org/
Pandas, at least v2.1.4	McKinney^15^	https://pandas.pydata.org/
Matplotlib, at least v3.8.0	Hunter et al.^16^	https://matplotlib.org/
Seaborn, at least v0.12.2	Waskom^17^	https://seaborn.pydata.org/
Plotly, v5.20.0	Plotly Technologies Inc.	https://plotly.com/python/
Pillow (PIL fork), at least v10.3.0	Murray et al.^18^	https://python-pillow.org/
MATLAB, at least 2017a for AQuA, at least 2022b for AQuA2	The MathWorks, Inc.	https://www.mathworks.com/products/matlab/
Curve Fitting Toolbox, v23.2	The MathWorks, Inc.	https://www.mathworks.com/products/curvefitting/
Image Processing Toolbox, v23.2	The MathWorks, Inc.	https://www.mathworks.com/products/image-processing/
Statistics and Machine Learning Toolbox, v23.2	The MathWorks, Inc.	https://www.mathworks.com/products/statistics/
AQuA	Wang et al.^9^	https://github.com/yu-lab-vt/AquA/
Fiji	Schindelin et al.^19^	https://fiji.sc/
STARDUST	-	https://github.com/papouinlab/STARDUST/

## Step-by-step method details

This section describes the step-by-step process for 1) Part 1: image preprocessing (including motion correction and noise filtering), 2) Part 2: active pixels detection using AQuA, 3) Part 3: map of region-of-activity (ROA) generation, 4) Part 4: time-series data extraction, 5) Part 5: cell mask acquisition, and finally 6) Part 6: signal detection and feature extraction with a Python pipeline. Together, this permits the analysis of calcium signals extracted from raw recordings, illustrated using two-photon laser scanning microscopy (2-PLSM) recordings of the calcium sensor lck-GCaMP6f and static tdTomato reference marker expressed in astrocytes (AAV5-gfaABC1D::lck-GCaMP6f, AAV5-gfaABC1D::tdTomato, micro-injected at P70) in acute hippocampal slices obtained from adult mice.

**Figure 1:**
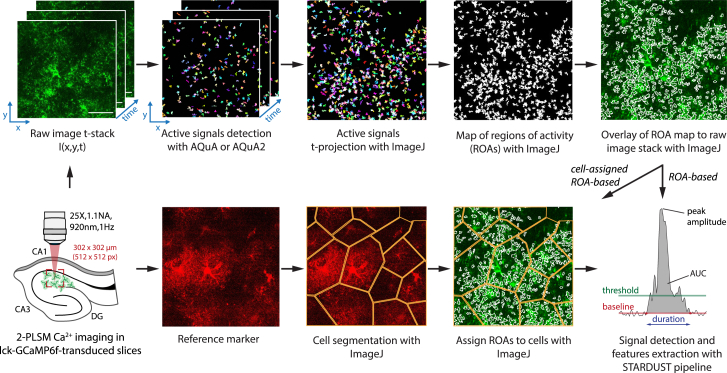
Illustration of the STARDUST workflow Note that STARDUST permits three types of analysis (see main text): ROA-based analysis, wherein the cell boundaries and identity of the cells to which individual ROAs belong are ignored (top raw), cell-based analysis, consisting of overall fluorescence analysis within entire cell boundaries with no ROA definition, and cell-assigned ROA analysis, wherein ROA-based analysis is combined with cell-segmentation to yield a readout of average ROA activity within individual cells. Scale bar: 50 μm.

**Figure 2:**
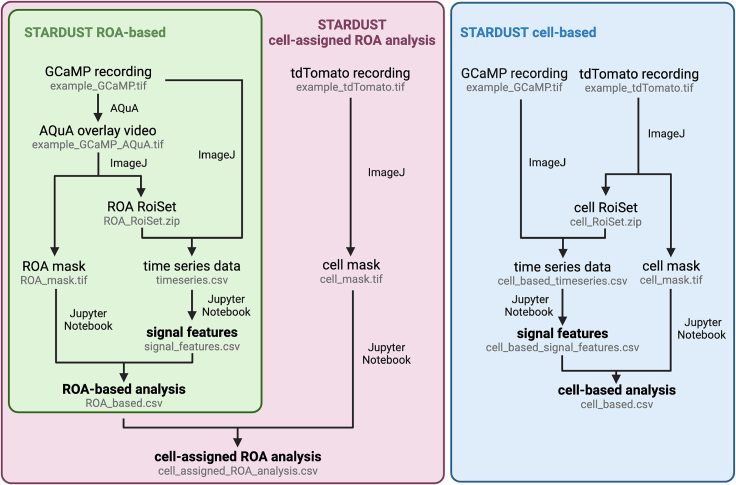
Flowchart for all three version of STARDUST, indicating the major steps, file outputs (in gray) and software required

***Note:*** The goal of STARDUST is to analyze regional activity in astrocytes (Figure 1). However, to allow a more versatile use of the pipeline, we implemented three versions of STARDUST best suited for three types of analysis (1) STARDUST cell-based analysis (2) STARDUST ROA-based analysis, and (3) STARDUST cell-assigned ROA analysis (Figure 2). Each type of analysis comprises its own code for Part 6: Signal detection and feature extractions.

### STARDUST cell-based version

If responses from entire cells are of interest, i.e., overall fluorescence within entire cells, use STARDUST cell-based analysis by following these steps (in this order): 1) Part 1: Image preprocessing, 2) Part 5: Cell mask acquisition, 3) Part 4: Time-series data acquisition from cell selection, 4) Part 6: Signal detection and feature extraction. Use the with Jupyter Notebook file STARDUST cell-based.ipynb when performing Part 6. Parts and steps required for this version of analysis are summarized in Table 1.

**Table 1:** Input and output files for STARDUST cell-based analysis pipeline

Part & step	Software	Input	Input format	Output	Output format
part 1 steps 1–3	ImageJ	Raw GCaMP recording and raw tdTomato recording	tif	N/A	N/A
part 1 step 2b (optional)	MATLAB NoRMCorre	Raw GCaMP recording.	tif	Motion-corrected GCaMP recording.	tif
		Raw tdTomato recording.	tif	Motion-corrected tdTomato recording. Same motion correction from the GCaMP recording should be applied.	tif
part 5 steps 22–25	ImageJ	Raw tdTomato recording or motion-corrected tdTomato recording.	tif	Cell RoiSet. A ImageJ zip file containing all cells delineated.	zip
				Cell mask.	tif
part 4 steps 18–21	ImageJ	Raw GCaMP recording or motion-corrected GCaMP recording.	tif	Time series csv file. Extracts fluorescence intensity time-series from each cell.	csv
		Cell RoiSet.	zip		
part 6 steps 26–36, ∗, 41	Jupyter Notebook STARDUST cell-based	Time series csv file.	csv	Metadata. Metadata associated with the recording and analysis.	csv
				dff traces. The ΔF/F_0_ traces from the current analysis.	csv
		Cell mask.	tif	Signal features. Each row represents one active signal detected by STARDUST.	csv
				Cell based analysis. Each row represents one cell and its summarized signal features.	csv

For details in part 6, read instructions from the steps specified in the table and from the Jupyter Notebook STARDUST cell-based.ipynb.

### STARDUST ROA-based version

If regional activity in micro-domains is of interest regardless of cell correlations (i.e., cell boundaries and identity of the cells to which individual ROAs belong are not relevant), use STARDUST ROA-based analysis: simply skip “Part 5: acquiring cell mask” of this protocol and use the Jupyter Notebook STARDUST ROA-based.ipynb when performing Part 6. Parts and steps required for this version of analysis are summarized in Table 2.

**Table 2:** Input and output files for STARDUST ROA-based analysis pipeline

Part & step	Software	Input	Input format	Output	Output format
part 1 steps 1–3	ImageJ	Raw GCaMP recording and raw tdTomato recording	tif	N/A	N/A
part 1 step 2b (optional)	MATLAB NoRMCorre	Raw GCaMP recording.	tif	Motion-corrected GCaMP recording.	tif
part 2 steps 4–13	MATLAB AQuA	Raw GCaMP recording or motion-corrected GCaMP recording.	tif	AQuA overlay video. Contains colorful ROAs detected from AQuA.	tif
				AQuA MATLAB file. MATLAB project file in case output parameters need to be changed later.	mat
part 3 steps 14–17	ImageJ	AQuA overlay video.	tif	ROA RoiSet. A ImageJ zip file containing all ROAs detected from AQuA.	zip
				ROA mask.	tif
part 4 18–21	ImageJ	Raw GCaMP recording or motion-corrected GCaMP recording.	tif	Time series csv file. Extracts fluorescence intensity time-series from each ROA.	csv
		ROA RoiSet.	zip		
part 6 26–36, ∗, 41	Jupyter Notebook STARDUST ROA-based	Time series csv file.	csv	Metadata. Metadata associated with the recording and analysis.	csv
				dff traces. The ΔF/F_0_ traces from the current analysis.	csv
		ROA mask.	tif	Signal features. Each row represents one active signal detected by STARDUST.	csv
				ROA based analysis. Each row represents one ROA and its summarized signal features.	csv
				ROA summary. Type of ROA and count based on pharmacology application and calcium activity.	csv

For details in part 6, read instructions from the steps specified in the table and from the Jupyter Notebook STARDUST ROA-based.ipynb.

### STARDUST cell-assigned ROA analysis version

If regional activity is to be combined with cellular correlations (i.e., cell-segmentation will be required for a readout of average ROA activity within individual cells), follow the steps below as they are described and use Jupyter Notebook file STARDUST.ipynb to perform Part 6. Parts and steps required for this version of analysis are summarized in Table 3.

**Table 3:** Input and output files for STARDUST cell-assigned ROA analysis pipeline

Part & step	Software	Input	Input format	Output	Output format
part 1 steps 1–3	ImageJ	Raw GCaMP recording and raw tdTomato recording	tif	N/A	N/A
part 1 step 2b (optional)	MATLAB NoRMCorre	Raw GCaMP recording.	tif	Motion-corrected GCaMP recording.	tif
		Raw tdTomato recording.	tif	Motion-corrected tdTomato recording. Same motion correction from the GCaMP recording should be applied.	tif
part 2 steps 4–13	MATLAB AQuA	Raw GCaMP recording or motion-corrected GCaMP recording.	tif	AQuA overlay video. Contains colorful ROAs detected from AQuA.	tif
				AQuA MATLAB file. MATLAB project file in case output parameters need to be changed later.	mat
part 3 steps 14–17	ImageJ	AQuA overlay video.	tif	ROA RoiSet. A ImageJ zip file containing all ROAs detected from AQuA.	zip
				ROA mask.	tif
part 4 steps 18–21	ImageJ	Raw GCaMP recording or motion-corrected GCaMP recording.	tif	Time series csv file. Extracts fluorescence intensity time-series from each ROA.	csv
		ROA RoiSet.	zip		
part 5 steps 22–25	ImageJ	Raw tdTomato recording or motion-corrected tdTomato recording.	tif	Cell RoiSet. A ImageJ zip file containing all cells delineated.	zip
				Cell mask.	tif
part 6 steps 26–41	Jupyter Notebook STARDUST	Time series csv file.	csv	Metadata. Metadata associated with the recording and analysis.	csv
				dff traces. The ΔF/F_0_ traces from the current analysis.	csv
		ROA mask.	tif	Signal features. Each row represents one active signal detected by STARDUST.	csv
				ROA based analysis. Each row represents one ROA and its summarized signal features.	csv
		Cell mask.	tif	Cell-assigned ROA analysis. Each row represents one cell and its summarized signal features.	csv
				ROA summary. Type of ROA and count based on pharmacology application and calcium activity.	csv

For details in part 6, read instructions from the steps specified in the table and from the Jupyter Notebook STARDUST.ipynb.

### Part 1: Image preprocessing

**Timing: >15 min**

This step includes recording quality checks and motion correction (time needed for this step will vary depending on the recording quality, whether pre-processing is carried out and with users’ experience).

***Note:*** Because astrocyte Ca^2+^ activity occurs predominantly in small nano/micro-domains^4^^,^^5^^,^^6^^,^^7^ that are both confined and difficult to tie to any anatomically defined sub-cellular structure, astrocyte Ca^2+^ recordings and subsequent analysis do not tolerate spatial drift. We thus strongly recommend applying a motion correction against noticeable drift in the recording. If permitted, a second-channel recording of a static cell marker (e.g. tdTomato) can be used as a reference for cell registration.

1. Open the raw calcium recording tiff image file in ImageJ (e.g., example_GCaMP.tif in our example data).
2. Play the image stack and check the following:
  a. Recording quality.
    i. Open *B&C window* from *Image > Adjust > Brightness and Contrast.* Click “Auto” or adjust the slide bars to tune the contrast.
      ***Note:*** A good recording should have clear and bright signals that stand out from the background noise. For reference, please refer to supplement Methods video S1 for an example of a recording with good viral expression and low background noise, and supplement Methods video S2 for an example of a recording with low fluorescence (likely due to poor viral expression) and high background noise.
      Methods video S1. High signal to noise recording with good viral expression, related to Part 1 step 2a Recording was obtained from Bruker Ultima 2pPlus with Nikon 25X, 1.10NA objective, 1.6X digital zoom, laser power at 37.5 mW, recording rate at 1 Hz, recording depth at 40 μm.
      Methods video S2. Low signal to noise recording from hippocampal CA1 region with poor viral expression, related to Part 1 step 2a Recording was obtained from Bruker Ultima 2pPlus with Nikon 25X, 1.10NA objective, 1.6X digital zoom, laser power at 37.5 mW, recording rate at 1 Hz, recording depth at 40 μm.
  b. Shift during recording and registration.
    i. Identify a marker, such as a cell body or a blood vessel, as a reference to check x, y drift.
      ***Note:*** If there is significant drift over time, i.e. the x, y location of obvious cell markers shifts away from their original position, the recording will require a registration. Compare the last frame to the first frame to assess the extent and direction of the drift. If the recording has a significant x, y shift, NoRMCorre (https://github.com/flatironinstitute/NoRMCorre) is a great MATLAB-based tool for non-rigid motion correction of calcium imaging data. However, due to the highly diverse nature of calcium activity in astrocytes, these tools occasionally fail to achieve an adequate registration. Therefore, the registered recording should be carefully examined before proceeding to the next step. Overall, an excessive x, y drift means that the data might simply need to be discarded from further analysis.
      ***Note:*** Although NoRMCorre works well in our hands for correcting x, y shifts, it often creates artifacts along the edges of the recording, which will be mistaken for aberrant active pixels in the following step. Please see notes in step 8 and step 14 to avoid generating aberrant ROAs when motion correction is applied.
    ii. Proceed similarly to assess vertical drift (z-axis).
      ***Note:*** A noticeable shift along the z-axis means that the fluorescence data are obtained from inconsistent focal plans over time. Since this cannot be corrected, we suggest removing the recording from analysis. A significant z-shift is one where initial anatomical features (soma, branches, blood vessel) are lost from the field of view over time.
3. Check the recording to assess overall fluorescence change by *Image > Stacks > Plot Z-axis Profile* to plot mean fluorescence in the entire field of view over time.

***Note:*** This plot helps users gauge the recording quality and identify epochs. Overall gradual increases or decreases in fluorescence intensity can come from a change in activity or photo-bleaching. In the absence of pharmacological manipulations, this might be indicative of a poor or unstable recording conditions. On the other hand, this is particularly useful when pharmacological treatments were performed as this might facilitate pinpointing the time of drug wash-in.

### Part 2: Active pixels detection using AQuA or AQuA2

**Timing: 30 min–1 h**

This section uses AQuA, a publicly available software package, to identify active pixels that are above noise threshold. All supra-threshold active pixel will be color-labeled after analysis. If running STARDUST cell-based analysis, this part can be skipped.

***Note:*** At the time of revision of this Protocol, a preprint describing AQuA2 has been released.^20^ AQuA2 is an updated version of AQuA with a greatly improved processing time. Importantly, the AQuA2 interface is very similar to that of AQuA. Even though it has not yet been peer-reviewed and user-vetted, we expect that many users will rapidly adopt AQuA2. Hence, while our protocol is built on AQuA, we have confirmed that the STARDUST workflow is compatible with AQuA2 outputs and provide notes to that effect in the following steps.

4. Launch MATLAB.
  a. In the “Current Folder” toolbar, navigate to the folder where the file “AQuA-master” is saved.
  b. Add the “AQuA-master” folder to the working directory by right clicking the folder name in the “current folder” window.
  c. Select “Add to Path” and “Selected Folders and Subfolders”.

***Note:*** If using AQuA2, MATLAB versions no older than 2022b are recommended. In AQuA2, navigate to the folder named “AQuA2-main” instead of the step above.

**Figure 3:**
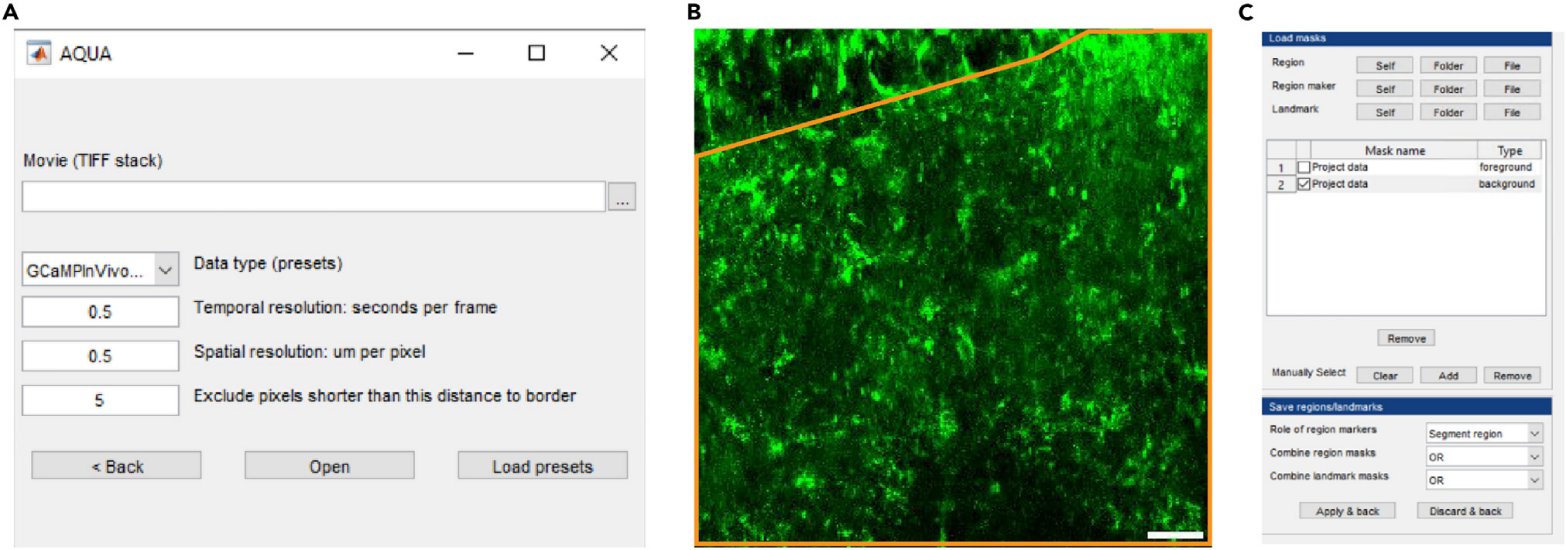
Detecting active voxels using AQuA (A) The AQuA launch window. (B) An area of interest for subsequent analysis is drawn (orange) on the image stack reference. Scale bar: 20 μm. (C) The mask builder interface.
6. Input the image file to analyze by clicking the three dots on the right.
7. In the “Data type” drop-down menu, select the type of imaging condition in the corresponding data file. In our example images, data are collected from acute brain slices with lck-GCaMP6f calcium indicator, and we choose “GCaMPExVivoLck”.

***Note:*** Present analysis parameters are determined according to the Data type selected. Data types include GCaMP *in vivo* (lck & cyto), GCaMP *ex vivo* (lck & cyto), GluSnFR, noisy GCaMP etc. Presets can be modified in the “parameters 1.csv” file in the “cfg” folder of “AQuA-master”. Please refer to the AQuA documentation on the GitHub page to determine the “Data type” that most closely matches your dataset and instructions on how to modify them if needed.

***Note:*** In AQuA2, the data type drop-down menu includes “default” and “long-duration signal”. Select the data type that matches your input data. Presets can be modified in the “parameters.csv” in the “cfg” folder of “AQuA2-main”.

5. Launch AQuA by entering “aqua_gui” in the Command window. A popup window of AQuA with “New project” and “Load existing” will appear. Click “New project” and a field for file selection and parameter options will show as in Figure 3A.

8. After selecting the data type, pre-selected values will be automatically filled for the three relevant parameters at the bottom.
  a. Edit the temporal resolution and spatial resolution according to recording conditions.
  b. For “Exclude pixels shorter than this distance to border”, change to “0” to ensure that the output file has the exact same canvas size as the raw file.

**CRITICAL:** If no motion correction is applied, setting exclude pixels at 0 is critical for accurately overlay the map of ROA back onto the raw file in step 18.

**CRITICAL:** If a motion-corrected recording is used, because of possible edge aberrations from correction (see note in step 2b), “Exclude pixels shorter than this distance to border” should be set to non-zero values (Figure 3A). For minor motion correction, set “exclude pixels shorter than this distance to border” between 2 and 5.

9. Click “Open” to proceed to the analysis window.
  ***Note:*** In AQuA2, there is a pre-processing window that allows users to conduct motion correction and remove noise. Careful examinations of the motion-corrected and noise-removed results are encouraged given the complex nature of astrocyte calcium as stated in step 2b.
  ***Optional:*** Remove untargeted areas in the field of view in AQuA analysis. The top left box “Direction, regions, landmarks” includes functions such as defining cell boundary or areas, or creating an anatomical mask. This step can be applied to exclude undesired areas of recording or restrict the analysis to areas of interest. Specifying areas of interest reduces the processing time in AQuA. Here we provide an example of how to select a specific area for analysis. In this example, we are interested in analyzing the area outlined in yellow (Figure 3B) and would like to exclude other areas from further analysis.
  a. Select “Mask builder” in the box “Direction, regions, landmarks”.
  b. In the pop-up window, select “Self” under “Region” (Figure 3C). “Self” means the selected recording will be used as a reference to build the mask. After clicking “Self”, some areas are automatically selected if the pixels are above a default intensity threshold.
  c. To build a new mask that outlines the area of interest, clear the default areas by clicking “Clear”.
  d. Click “Add” to draw the area of interest by hand under the “Manually Select” section.
  e. Click “Apply & back”. If successfully selected, the movie in the AQuA main window will have the area(s) outlined and numbered. Please refer to the AQuA user guide for other ways to determine desired areas of interest in the field of view.
10. Set the parameters for detection of active pixels.
  a. In the detection window on the left, check the box “skip steps 2, 3, 4” to detect active voxels without further processing and merging.
    **CRITICAL:** When including steps 2, 3, and 4, the full-length AQuA platform assigns active voxels that coherently occur in spatially connected regions into “events” based on their spatio-temporal properties and assumptions about event propagation that are not necessary in STARDUST. Instead, the STARDUST pipeline analyzes raw active pixels.
    ***Note:*** In AQuA2, there is no box for “skip 2,3,4”. Instead, users need to run each step to disable them individually.
  b. Set the intensity threshold scaling factor, smoothing, and minimum size for active pixels detection.
    ***Note:*** Default setting provides a good starting point for parameter selections. However, to ensure low false detection rate, it is crucial to optimize parameters based on recording properties. For example, the intensity scaling factor can be fine-tuned by comparing the true signals in the input recording with the color-labeled signals in the output. Increase the scaling factor if excessive noise is detected and decrease it if true signals are omitted. The minimum size can be determined by finding the smallest pixel groups that give meaningful signals, i.e., that exhibit real signals, not noise, in the raw images. In our analysis, we use an intensity threshold scaling factor of 2.5, a smoothing (sigma) of 0.5, and a minimum size 10 for processing. Please refer to the AQuA user guide for more detailed explanation of these parameters.
    ***Note:*** Because AQuA2 uses a different framework, parameters suggested above might not be the optimal for AQuA2. However, the same principles can be used for optimization.
11. Click “Run all steps” to run the pipeline. A proof-reading window and export window will appear once the software finishes processing.

***Note:*** The processing time depends on the size, quality of the recording, and computer hardware. For a 600 s recording with a size of 512 × 512 pixels, the processing time is ∼30 min.

***Note:*** The processing time of AQuA2 is significantly shorter than AQuA, especially for large dataset. For a 600 s recording with a size of 512 × 512 pixels. The processing time is ∼8 min.

**Figure 4:**
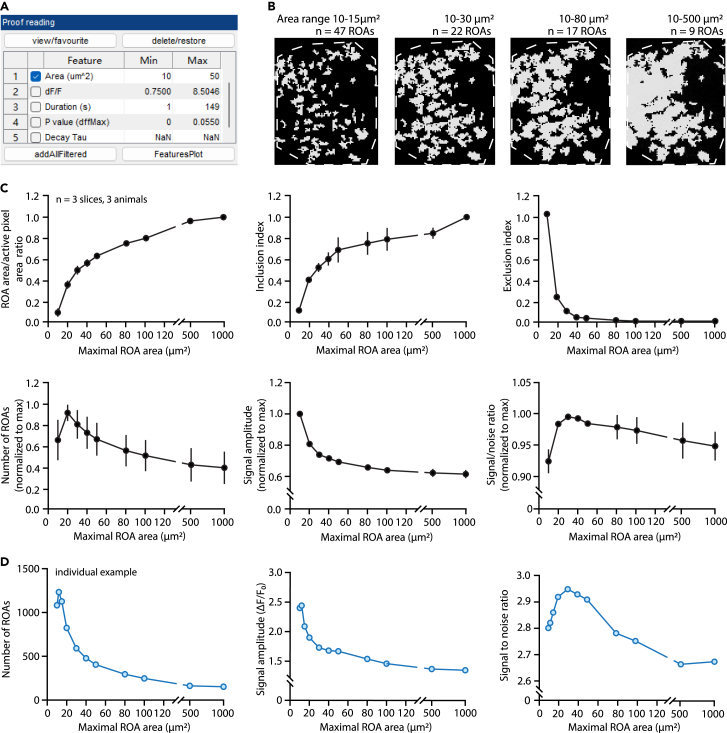
Determining the adequate range of ROA sizes (A) “Proof reading” window in AQuA for selecting appropriate filter criteria of the output movie. (B) Effect of the min and max cutoff values for the “Area” parameter on the map projection of active zones (grayed areas) over a representative astrocyte (cell boundary is denoted with dotted outline). The number of ROAs eventually obtained with each max area cutoff is indicated. Note that as active zones enlarge, they tend to merge, resulting in larger but fewer ROAs (also see below). The recording was acquired on Nikon A1R microscope. (C) Effect of the max Area cutoff on diverse parameters. The inclusion index reflects how the map of ROAs, for a given max area cutoff value, successfully captures the large active pixel patches that might be excluded by this setting. It is measured as the percentage of area of the excluded large pixel patches that overlaps with the final map of ROAs. The exclusion index measures the number of active pixel patches that have no pixels included in the final map of ROAs. Note that the optimal cutoff should be determined by considering all factors. In our experience, cutoffs that give high number of ROAs or high signal to noise ratio are usually good candidates. Data are shown as mean ± sem. Recordings were acquired on Nikon A1R microscope. (D) Example of the effect of max area cutoff on the final number of ROAs, average signal amplitude and signal to noise ratio for an individual experiment. Note how peaks, optima, or minima may occur for different max area values across experiments (compared to C). Recording was acquired on Nikon A1R microscope.
  b. Optimize the range of areas to capture as many regions of activity (ROAs) and signals as possible in later steps.

***Note:*** We recommend testing out different ranges of area min and max cutoffs because the adequate range will depend on the imaging conditions and image stack quality (see Figure 6). Too stringent a filter criterion will lead to the loss of active regions, while too loose a criterion will combine/merge adjacent regions into a single ROA leading to a loss of spatial resolution and a decrease in signal to noise ratio as illustrated in Figure 4 (troubleshooting 1).

***Note:*** We recommend keeping the max area cutoff value within a narrow range (in our case 20 μm^2^–40 μm^2^) across the same dataset but adjusting it as needed for each individual recording. The min area cutoff value is usually the same as in the detection setting.

***Note:*** For the same reason stated in the note of step 10b, optimized range areas might be different for AQuA2. AQuA2 users can apply the same principles to optimize the area range cutoffs.

12. Set the filter condition for the output file. This step is crucial for Part 3 ROA map acquisition because different filter condition determines a different ROA map.
  a. Select the parameter “Area”, which is the size of the active zones in μm^2^, as the filter criteria by checking the adjacent box in the “Proof reading” window (Figure 4A).

13. Export analysis results from AQuA.
  a. Export the feature overlay video.
    i. In the “Layers” window on the right under the “Movie brightness/contrast”, drag the range slider all the way to the left to set brightness to zero for the raw recording.
      ***Note:*** In AQuA2, there are “Min” and “Max” range sliders. Drag only the “Min” slider to the right to set the brightness to zero for the raw recording. The “Max” slider can be at a random position.
    ii. Next, in the “Feature overlay” section, in the “Layers” window, drag the slider to the right to set the brightness of the feature overlay video to max.
      ***Note:*** In AQuA2, drag the “colorful overlay brightness” range slider all the way to the right to set the brightness of the overlay to max.
      ***Note:*** (i) and (ii) ensure that raw movie pixels are removed and only the color-labeled overlay is included in the output movie. This is critical because raw movie pixels might interfere with ROAs identification from the t-projected image in steps 14–15.
    iii. In the “Export” window on the left, check only the “Movie with overlay” box to export the movie overlay. Click “Export/Save”.
    iv. Type in your desired suffix for the output file name. Navigate to the target folder to save the feature overlay video. The feature overlay video will be saved in the tiff format.
  b. Save the processed AQuA MATLAB file.
    i. Select “Events” in the “Export” window and save to the directory.
      ***Note:*** Saving the AQuA MATLAB file allows for adjusting filtering criteria and output new overlay movies later if needed.
      ***Note:*** To later access a saved AQuA MATLAB file, select “Load existing” after starting an aqua_gui window in step 5.

### Part 3: Acquisition of the map of regions of activity (ROAs)

**Timing: 5 min**

This section describes how to generate the map of regions of activity (ROAs) from the AQuA output movie using ImageJ. We define a ROA as the aggregate spatial footprint formed by the t-projection of a confined ensemble of active pixel patches. As a result, 1) a ROA is a spatial domain within which at least one active pixel patch occurs during a recording, 2) active pixel patches within a ROA do not need to occupy the entire spatial footprint of the ROA, 3) the footprints of two active pixel patches from a same ROA do not necessarily overlap, as it is the aggregate footprint of all active pixel patches that defines the boundaries of the ROA, 4) any two given ROAs are spatially unconnected, and 5) the constellation of ROAs of a given cell represents a map of spatially independent zones of activity within that cell. The activity of two contiguous ROAs is assumed to be independent (however, by vertue of the map of ROA acquisition process, concomitant signals may be observed in several contiguous ROAs). In total, the advantages of this analysis method are that it is unbiased, semi-automated, comprehensive, and solely activity-based in that it is decoupled from (but compatible with) morphological constrains or cell-based criteria. Note that this part is required for STARDUST ROA-based analysis and cell-assigned ROA analysis, but should be skipped for cell-based analysis.

14. Acquire the map of ROA by performing a time projection on the output file in tiff-format from AQuA. Open the file in ImageJ. Click *Image > Stacks > Z Project > Max intensity* to generate a t-projected image.

**CRITICAL:** If a motion-corrected recording is used, and “Exclude pixels shorter than this distance to border” was set to a non-zero value for processing with AQuA in step 8 (Figure 3A), the AQuA output movie will be cropped. For example, for a recording with 512 × 512 pixels dimension, if “Exclude pixels shorter than this distance to border” is set to 2, pixels within 2 pixels distance to the border will be ignored, and AQuA will analyze and output the 508 × 508 pixels from the center of the video. To restore the canvas size to ensure that the ROAs can be mapped back onto the GCaMP recording file to extract time series, adjust the image size by *Image > Analysis > Canvas size*. Change to 512 × 512 pixels and leave the position at “Center”.

15. Assign ROI numbers to each ROA for analysis in ImageJ.
  a. Convert the t-projected ROA map to binary image by *Process > Binary > Make Binary.*
    ***Note:*** ImageJ does not have a “ROA” manager, instead we use the ROI manager for any selected regions of interest, whether ROAs or cells (see Part 5). Beware of possible confusions in the subsequent text.
  b. Select *Analyze > Analyze particles.* Set the size to 0-infinity and leave the box of “Pixel units” unchecked. Set the circularity to 0.00–1.00. Select “Nothing” from the “Show” drop-down mean. Check the boxes for “Include holes”, “Add to manager” and “Composite ROIs”. Leave the boxes for “Display results”, “Exclude on edges”, “Summarize” and “Overlay” unchecked. Click “OK”.
    ***Note:*** In the ROI manager, each ROA is assigned a number and highlighted with colored boundaries on the image. The total number of ROAs can corresponds to the last number in the ROI manager.
    ***Note:*** Large, connected ROAs may arise if the filtering criteria in step 12 are not optimized (See Figure 4, troubleshooting 1).
16. Save all the ROAs (ROI selections). In the ROI manager, select all ROAs and right click to save as a ROA_RoiSet.zip file. This file can be reloaded into ImageJ if the ROA map needs to be adjusted later.
17. Generate a binary ROA mask.
  a. Select all the ROAs in the ROI manager (Ctrl+A), right click and select “OR (Combine)”, then click “Add [t]” in the ROI manager. This generates a new ROI that combines all the ROAs.
  b. Select this ROI and then go to *Edit > Selection > Create Mask.* A binary mask with all of ROAs shown in pixel value 255 (default as white) and background shown in pixel value 0 (default as black) is generated. Save this mask as ROA_mask.tif under the same file folder as all other files by clicking *File > Save as > Tiff* file.

### Part 4: Time-series data acquisition

**Timing: 3 min**

This section extracts fluorescence intensity time-series from each ROA in the ROA map.

18. In ImageJ, open the original or motion-corrected GCaMP recording tiff file. Also open the ROA_RoiSet.zip file generated from step 16. Overlay the ROA selections onto the original file by clicking “Show All” in the ROI manager.
19. To measure the fluorescence intensity of each selected ROA, set measurement item in *Analyze > Set measurements.* Check the box “mean gray value” and leave the other boxes unchecked.

***Optional:*** The area of each ROA can also be obtained with the same method by checking the box “Area” instead. This is of interest when comparing ROA features (e.g., numbers, size) across different conditions (see Figure 6 for example). Note that ROA size for each ROA is also extracted later in part 6.

20. To measure the fluorescence intensity across all frames, select all the ROAs in the ROI manager, right click and select “multi measure”. In the next popup window, select “measure all slices” and “one row per slice”. A result window will then appear with the values of intensity for each ROA over all frames.
21. Save the result as timeseries.csv through *File > Save as* in the results window to the same folder where ROA mask was saved.

### Part 5: Cell mask acquisition

**Timing: 30 min**

This step serves to identify the cell boundaries in the field of view. Note that this step is required for STARDUST cell-based analysis and cell-assigned ROA analysis, but not for ROA-based analysis.

22. Obtain a reference image for cell boundaries in the recording field of view.
  ***Note:*** Because of the complex structure of astrocytes, determining the cell boundary for each cell is intrinsically challenging. We offer two suggestions to obtain a reference image for manually outlining cell boundaries.
  a. If multichannel recordings or images from a second, static marker were acquired, the reference image can be obtained from them. In our analysis, lck-GCaMP6f and tdTomato, used as a call marker, are co-expressed in astrocytes and imaged in the green and red channel respectively.
  b. If no static markers were used, a reference image can be obtained by generating a z-projection from the original t-stack recording by *Image > Stacks > z projection.*
    ***Note:*** Because baseline fluorescence in the lck-GCaMP6f is usually low, and because cyto-GCaMP6f primarily reveals astrocytes soma and branches, the arborization of individual astrocytes may not appear in full in either case. However, this might suffice to outline the general shape of astrocytes.
  c. If cell segmentation is of paramount importance, we strongly recommend using sparse expression of GCaMP6f (usually by reducing viral titer or tamoxifen dosage) and other cell segmentation tools such as Imaris.
23. Assign ROAs into their respective cells.
  a. In ImageJ, open the reference image and the ROA_RoiSet.zip file, and overlay the ROA selections onto the reference image by clicking “Show All”.
  b. Delineate cell boundaries with the “Polygon selections” tool, making sure to include all ROAs in their respective cell.
  c. Add each cell selection to the ROI manager by “Ctrl +t” or “Add” in the ROI manager.

**CRITICAL:** Because astrocyte domains have virtually no or minimal spatial overlap, cell boundaries should not touch each other. Two closest points from neighboring cells should be at least one pixel away, including diagonally (troubleshooting 2).

***Note:*** All ROAs should be captured within a cell boundary, since astrocytes tile the entire neuropil. It can be challenging to determine whether a set of processes/ROAs belongs to an adjacent astrocyte within the focal plane or to an astrocyte outside of the focal plane, by virtue of their 3D tiling. Here again, if this is an issue, see steps 22b and 22c above.

24. Select and delete all the ROAs in the ROI manager, leaving only the cell selections. Select all and right click to save as cell_RoiSet.zip.
25. Generate the cell mask.
  a. Select all the cell selections in the ROI manager, right click and select “OR (Combine)”, then “Add” to the ROI manager. This generates an ROI that includes all the cell selections.
  b. Select this ROI and then go to *Edit > Selection> Create Mask.* Save it as cell_mask.tif under the same file folder. This folder should now contain the cell mask tiff file (cell_mask.tif), the ROA mask tiff file (ROA_mask.tif), and the time series csv file (timeseries.csv).

***Note:*** To this point, the ROA mask (ROA_mask.tif) and cell mask (cell_mask.tif) and the fluorescence time-series data (timeseries.csv) have all been generated. These three input files are needed for downstream signal detection and analysis for the cell-assigned ROA analysis. If performing ROA-based analysis, the ROA mask (ROA_mask.tif) and the fluorescence time-series data (timeseries.csv) are required. If performing cell-based analysis, the cell mask (cell_mask.tif) and the fluorescence time-series data (timeseries.csv) are required.

### Part 6: Signal detection and feature extraction

This section introduces an interactive and user-friendly Jupyter Notebook Python script for signal detection and analysis. Here, we take users though the “STARDUST cell-assigned ROA analysis”, which comprises all steps common to all three versions of STARDUST (Figure 2; Table 3, and code annotations). Code segments shown here thus come from the “STARDUST.ipynb” version, which includes signal preprocessing, baseline fluorescence (F_0_) determination and signal detection, signal feature extraction, cell assignment, ROA-based analysis, cell-assigned ROA-based analysis, and data output.

***Note:*** When running the analysis pipeline, the script will take users through the analysis flow semi-automatically, with interactive prompts, stopping at steps that require user inputs or provide output graphs for visual inspection. To streamline the process, the Jupyter Notebook is equipped with custom-defined functions stored in util.py. All scripts are available on https://github.com/papouinlab/STARDUST.

***Note:*** This section provides explanations for the inputs required by each code block, the underlying computation, and the outputs they give. For more detailed explanation of arguments and outputs, users can use the help documentation for each function by executing help(***function_name***) in the interactive environment. Notes on how to optimize key parameters are also detailed in this section.

**CRITICAL:** Unless explicitly noted, **the users do not need to edit the content of the code**.

***Note:*** Code blocks are referred to as “cells” in the Jupyter Notebook ecosystem. To avoid confusion, we will be referring to these as code blocks in this protocol.

***Note:*** In the STARDUST pipeline, each non-optional code block needs to be run before executing the next block. To execute a code block, select it by clicking anywhere inside it, and click the “Run” button on the top of the Jupyter Notebook page or use Shift + Enter. There will be a set of empty brackets [ ] on the left side of each code block. When the code is running, the brackets will show an asterisk sign [∗]. When the code completes running, a number will show up in the bracket, marking the order of executed code blocks.

***Note:*** Some code blocks, upon running, will prompt users for input in a text box under the code block. Input the prompted information and press Enter to proceed to the next step.

26. Download the STARDUST repository from GitHub. On the top right of the repository webpage, click the green “Code” button, click “Download ZIP” to a local directory. Extract all files.
  ***Note:*** Because online Jupyter Notebook navigates to the C Drive in Windows system by default, we recommend downloading the Zip file into the C Drive.
  ***Optional:*** Although it is possible to run STARDUST in the base environment in Anaconda, we recommend setting up a designated environment for STARDUST to avoid module version conflicts with other pipelines. We provide a YAML file with required software and module versions for STARDUST in the GitHub repository. YAML is a human-friendly data serialization language that can be directly read by the Anaconda navigator.
  a. To import the STARDUST environment in Anaconda, open the Anaconda navigator and click “Environments” in the left panel to navigate to the environments page.
  b. In bottom of the middle panel, click the “Import” button. A “Import Environment” window will appear and “Local drive” will be selected by default under “Import from”.
  c. Click the file icon next to the empty text box under “Local drive”, navigate to the STARDUST-main folder downloaded earlier in this step, select the “STARDUSTreq.yml” file and click “Open”. The text box should now show the path to the YAML file.
  d. Change the “New environment name” if needed.
  e. Click the “Import” button.
27. Launch the Jupyter Notebook version of choice from the Anaconda navigator home page. This will open a browser tab showing the Notebook Dashboard. The Dashboard is similar to a file explorer.
  a. Navigate to open the STARDUST GitHub folder “STARDUST-main”.
  b. Select and open one version of .ipynb file that matches the type of analysis. If performing STARDUST cell-based ROA analysis, open “STARDUST.ipynb”. If performing STARDUST ROA-based analysis, open “STARDUST ROA-based.ipynb”. If performing STARDUST cell-based analysis, open “STARDUST cell-based.ipynb”.
28. Execute the following code block by clicking “Run”, which will import modules and custom-defined functions.

>import pandas as pd, numpy as np, seaborn as sns, scipy, matplotlib, PIL

>from src.STARDUST.util import ∗

29. Run the follow code block to check that the environment is equipped with the required version for modules used.

>check_modules()

***Note:*** For how to set up the environment, refer to the optional note in step 26.

30. Run the next code block to read in input files and information about the experiment. Upon running, the code will prompt users to input the path to the folder that contains all input files, the file names, and information about the experiment including timing of pharmacology application, frame rate, and spatial resolution of the recording. Enter the information accordingly in the prompted text boxes.

***Note:*** To find the path to a folder in a Windows system machine, navigate to the folder, click on the address bar in the file navigator and copy the path. For example, the path to a folder named “example folder” under the Documents folder should look like “C:∖Users∖YourUserName∖Documents∖example folder”.

**CRITICAL:** The input files, including the time series csv file (step 21), the ROA mask tiff file (step 17), and the cell mask tiff file (step 25) must all be under the same folder.

>time_series_path, ROA_mask_path, cell_mask_path, output_path = prompt_input()

>drug_frame, frame_rate, spatial_resolution = get_metadata()

31. Run the code block below to read in the ROA mask and cell mask. This also labels ROAs and cells to allow ROA assignment to a specific cell.

>ROA_map_array, ROA_map_labeled, ROA_map_count = read_tif(ROA_mask_path, “ROA”)

>cell_map_array, cell_map_labeled, cell_count = read_tif(cell_mask_path, “cell”)

**CRITICAL:** This code block outputs the total number of ROAs or cells being analyzed. The number should match the number of ROAs or cells generated in earlier steps in ImageJ (troubleshooting 2).

***Optional:*** To visualize the ROA and cell map, run the following code block.

>visualize_map(ROA_map_array = ROA_map_array, cell_map_array = cell_map_array)

### Signal preprocessing

**Timing: 2 min**

This step provides signal smoothing and detrending. However, due to the enigmatic and dynamic nature of astrocyte calcium activity, signal preprocessing may alter or eliminate true features of calcium activity and therefore should be used with moderation, great caution and adequate rationale.

32. Run the code block to extract raw traces from the input file and generate filtered traces.

**Figure 5:**
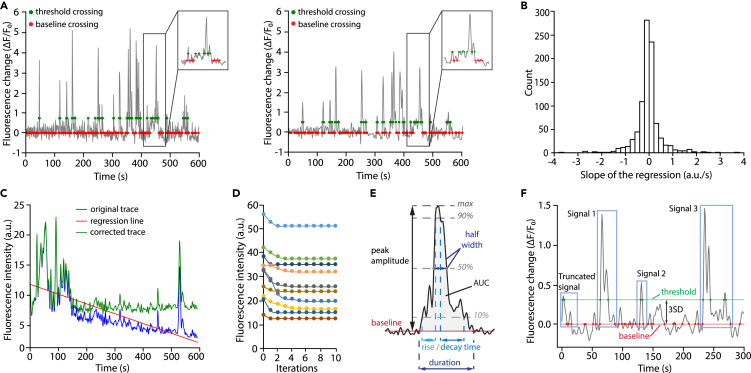
Signal detection and analysis (A) Left: raw ΔF/F_0_ trace; Right: ΔF/F_0_ trace filtered with a Butterworth filter. Note how after filtering the amplitude of signals is visibly diminished. Due to noise reduction, however, the 3SD threshold is also significantly lowered, resulting in minimal or no changes in signal detection. However, signal features might be affected. Red dots indicate where the traces cross F_0_. Green dots indicate where the traces cross the signal detection threshold. (B) Distribution of the linear regression slope from all ROA traces from a single recording. In a stable recording like this one (no or minima drift), the distribution is centered near 0 (see Figure 8A for the opposite example). (C) Example of drift correction. The downward drift in the original trace (blue) is corrected by subtracting a linear trend (red line). The corrected trace (green) is used for subsequent analysis. (D) Baseline fluorescence intensity (F_0_) of 10 individual time series over 10 cycles of iterations showing the rapid convergence of the baseline fluorescence determination process. In our hands, the baseline fluorescence value systematically converges within 5 iterations. (E) Schematic illustration of the main signal features and how they are measured. AUC: area under the curve; max: peak signal amplitude from baseline. (F) Illustration of a fluorescence time series extracted from a single ROA, showing baseline crossing points (red dots) and signal detection threshold crossing points (green dots) for 3 signals. Note the presence of a 'truncated’ signal due to the absence of an initiation point, which will be excluded from further analysis. 3SD: 3 standard deviations.

>raw_traces, filtered_traces = raw_to_filtered(time_series_path, order = 4, cutoff = 0.4)

***Note:*** To smooth fluorescence signals, a 4^th^ order low-pass Butterworth filter^21^ with a cutoff frequency of 0.4 Hz is applied to the signal traces, which filters the signal in a forward and backward direction to remove phase distortion. The low-pass filter is set to remove the high-frequency noise, while the cutoff frequency is set to ensure a minimal modification of the traces (Figure 5A). The order and cutoff frequency can be adjusted by specifying optional arguments *order* and *cutoff* in the ***raw_to_filtered*** function.

***Note:*** Users can choose to use the filtered traces or the raw traces in the following steps. By default, the filtered traces are used for further processing. To proceed with the raw traces instead, change “filtered_traces” to “raw_traces” in the code block in step 33 below.

33. Run the ***check_traces*** function to extract and output the total ROA number and number of frames. The result should match the number of ROAs from the ROA mask and the recording length in frames.

***Optional:*** Run the ***correct_shift*** function to visualize the slope distribution of all traces obtained using linear regression and probe potential fluorescence shift (Figure 5B). Note that the slope for each trace measures the degree of rise or decline of fluorescence over time. If the slope distribution from all traces is not centered around zero, this might indicate a drift or photo-bleaching during the recording in the entire field of view. This function then corrects linear trends by a default factor of 0.5 from the original traces (Figure 5C).

***Note:*** The degree of correction can be adjusted using the optional argument *correction_factor* which represents the percentage of linear trend to be subtracted in the function (troubleshooting 3).

>corrected_trace, reg = correct_shift(filtered_traces, correction_factor = 0.5)

>ROA_count, frame_count = check_traces(filtered_traces)

***Note:*** Linear regression is best used to remove a trend shared by all time-series in a recording (which is an indication that an overall drift occurred during the recording). By contrast, we advise that an upward or downward drift in only a sub-selection of ROAs might be indicative of a local or cell-specific change in baseline calcium levels, which is potentially biologically relevant and therefore should not be corrected. Additionally, beware that large signals tend to bias the linear regression calculation. Lastly, there exist other sophisticated methods for signal correction. Please select the optimal methods in specific conditions.

### Baseline fluorescence determination and signal detection

**Timing: 2 min**

Baseline fluorescence (F_0_) determination is a prerequisite for accurate signal detection and features extraction. F_0_ is determined by iterating a three-step process: 1) interim F_0_ determination, 2) above-threshold segments identification, 3) segments removal. During the first iteration, F_0_ is calculated as the average of the entire trace. Next, trace segments above the signal threshold (i.e., potential signals) are identified and removed. The interim F_0_ value at subsequent iterations is thus determined as the mean of the ‘signal-free’ trace. We find that F_0_ rapidly converges onto a value corresponding to the mean fluorescence of an entire trace devoid of signals exceeding the selected detection threshold (Figure 5D).

34. Run the following code block to determine F_0_. Input the desired number of iterations and signal threshold when prompted to.

>dff_traces, baselines, thresholds, signal_frames, signal_boundaries, signal_threshold = iterative_baseline(corrected_traces, baseline_start = 0, baseline_end = -1, include_incomplete = False)

***Note:*** Once the F_0_ is determined, the change in fluorescence intensity relative to F_0_ is calculated for each data point (ΔF/F_0_ trace) as: (F_(t)_ – F_0_)/ F_0_.

***Note:*** We find that convergence occurs within 5 iterations with no further improvement (and no deviation) with further iterations (Figure 5D). As for the signal threshold, we, and others, determine signal segments in traces based on the standard deviation (SD) in F_0_. We recommend setting the signal threshold as 2-to-3SD above F_0_.

***Note:*** A signal is defined by “F_0_ crossing points”, when the trace intersects the average F_0_, and “threshold crossing points”, when the trace intersects the signal threshold (Figure 5F). The bounds of a signal are thus the nearest F_0_-crossing points flanking one or more threshold-crossing points (Figure 5E).

***Note:*** Per this design, signals that lack an initiation or termination bound (i.e. one of the two F_0_ crossing points) will not be captured for analysis (Figure 5F, “Truncated signal”). This might result in some ROAs having no signals, even though they were derived from an active pixel patch. In occasions when truncated signals need to be included (for example, large signals with slow decay times that do not return to baseline during the recorded epoch) specifying the optional argument *include_incomplete* as True allows for extractions of available features (troubleshooting 4).

35. Run the next code block to visualize all ΔF/F_0_ traces of the current time-series file as a heatmap (also known as kymograph).

***Note:*** Each line of a kymograph shows the color-coded ΔF/F_0_ value of a single ROA over time.

>sns.heatmap(dff_traces, vmin = 0, vmax = (signal_threshold + 1) ∗ thresholds.mean(), xticklabels = 100, yticklabels = False, cmap = 'jet', square = True);

***Note:*** To facilitate visualization, we manually specify the optional arguments *vmin* and *vmax* that set the range of the color map. *vmin* specifies the lower bound of the color map. In our case we have set *vmin* at 0, meaning that ΔF/F_0_ values lower than 0 will show as dark blue. Similarly, *vmax* specifies the upper bound of the color map. We choose to set *vmax* at (signal_threshold + 1) ∗ average thresholds, so that signal segments with a ΔF/F_0_ value larger than *vmax* will be colored red. *vmin* and *vmax* can be adjusted as needed.

### Signal feature extraction

**Timing: 30 s**

36. Run the code block to extract the signal features with the ***analyze_signal*** function and generate a dataframe stored in signal_stats, with each row representing one active signal.

**Table 4:** Signal features output dictionary

Feature	Unit	Comment
ROA_ID	NA	ROA ID for the signal.
signal_start_frame	NA	The frame number of signal onset (F_0_ crossing).
signal_start_time	s	Time of signal onset (t = 0 being the start of the recording).
signal_end_frame	NA	The frame number at which the signal ends (F_0_ crossing).
signal_end_time	s	Time in seconds at which the signal ends.
peak_frame	NA	The frame number at which signal peak amplitude occurs.
peak_time	s	Time when signal peak amplitude occurs.
AUC	ΔF/F_0_·s	Area under curve for the selected signal, calculated using Simpson’s rule.
amplitude	ΔF/F_0_	Peak amplitude of the signal.
signal_to_noise	NA	Ratio of signal peak amplitude to noise peak amplitude of the baseline fluorescence of the selected ROA.
rise_time	s	Time elapsed between the 10% and 90% peak crossing points in the signal rise phase.
decay_time	s	Time elapsed between the 90% and 10% peak crossing points in the signal decay phase.
half_width	s	Time elapsed between the 50% peak crossing points in the rise phase and the 50% peak crossing points in the decay phase.
duration	s	Time elapsed between the signal initiation and signal termination.
inter_event_interval	s	Time, at the initiation point of a new signal, elapsed since the termination point of the previous signal in the same ROA. Returns “NA” if the signal is the first (or only) signal on the time-series.
epoch	NA	In experiments with pharmacology treatments, will return “baseline” if the signal peak occurs before the input drug_frame, and “drug” if it occurs after input drug_frame). Returns “NA” when input drug_frame was not inputted.

Also see Figure 5E.

>signal_features = analyze_signal(dff_traces, signal_frames, signal_boundaries, frame_rate, drug_frame)

***Note:*** These definitions in Table 4 depart slightly (but importantly) from those typically employed for neuronal recordings. For instance, in neuronal recordings, the decay time is often the time constant of the exponential fit applied to the “90%–10% of peak amplitude” portion of the trace, and the inter-event interval is determined from signal onset to signal onset. However, few astrocyte Ca^2+^ signals appear to display EPSC (excitatory postsynaptic current)- or EPSP (excitatory postsynaptic potential)-like kinetics. Additionally, in most Ca^2+^ recordings of astrocyte activity, the sampling rate (∼1 Hz) is often too low for an adequate exponential regression, and the duration of astrocyte Ca^2+^ transients are highly variable, prompting these minor differences.

### Cell assignment

**Timing: 30 s**

This step uses the two binary masks, ROA_mask.tif (step 17) and cell_mask.tif (step 25), to assign each ROA to the cell with which it overlaps. This step is applied to group ROAs of a given cell together and carry out subsequent cell-assigned ROA analysis.

37. Run the ***align_ROA_cell*** function to assign the correct cell IDs to each ROA, using the ROA and cell masks provided. The function also calculates ROA sizes.

***Note:*** ROAs that were not circled into any cells are assigned to “cell 0”.

>ROA_info = align_ROA_cell(ROA_map_labeled, cell_map_labeled, ROA_map_count, spatial_resolution)

38. Run the code block to add ROA ID, ROA size, and cell ID to the extracted signal feature data frame.

>signal_features = pd.merge(ROA_info, signal_features, on = 'ROA_ID', how = 'right')

### ROA-based analysis

**Timing: 1 min**

39. Run the next code block to perform the ROA-based analysis, which will summarize the averaged signal features per ROA (Table 4) and the ROA specific features (Table 5). This step also categorizes each ROA according to its activity in response to pharmacological treatments (when applicable).

**Table 5:** ROA-based analysis output dictionary

Output	Unit	Comment
ROA_ID	NA	ROA ID.
cell_ID	NA	Cell ID of selected ROA.
size_um2	μm^2^	Size of the selected ROA.
ROA_type	NA	ROA categorization based on its activity with regards to the drug_frame input. If signals were detected in the baseline and drug perfusion epochs of the time-series of the selected ROA, the ROA is categorized as “stable”. If the time-series contain signals during ‘baseline’ epoch but non during the ‘drug’ epoch, it is listed as an “off” ROA. If the time-series contains signals in the ‘drug’ epoch but none in the ‘baseline’ epoch, it is listed as “on” ROA. If no signals are detected, the ROA is listed as “inactive” (See troubleshooting 5 if a recording contains a high proportion of “inactive” ROAs). Returns “NA” if no value was inputted for drug_frame.
epoch	NA	Epoch from which average measurements were obtained.
signal_count	NA	Number of signals in the indicated epoch.
frequency_permin	min^–1^	Number of signals per min in the selected ROA during the indicated epoch.

>ROA_based, ROA_info = ROA_analysis(signal_features, ROA_info, frame_count, frame_rate, drug_frame, cell_segmentation = True)

### Cell-assigned ROA analysis

**Timing: 1 min**

40. Run the next code block to generate the cell-assigned ROA analysis.

>cell_assigned = cell_analysis(signal_features, ROA_info)

### Data output

**Timing: 30 s**

41. Run the next code block to output the analysis results. The results are compiled as pandas dataframes and can be exported as an Excel file with multiple datasheets or separate csv files by specifying the *save_as* argument as “excel” or “csv”.

***Note:*** Depending on the analysis results, the outputs include, but are not limited to: metadata containing important parameters such as the time of drug application, the list of ΔF/F_0_ traces for all ROAs, the list of ΔF/F_0_ traces averaged from ROAs per cell, the master sheet with all features analyzed for each signal, the master sheet for ROA-based analysis and the cell-based averaging across ROAs calculated from ROAs within respective cells.

>metadata = pd.DataFrame({'frame_rate': [frame_rate], 'spatial_resolution': [spatial_resolution],'drug_frame': [drug_frame], 'drug_time': [drug_frame/frame_rate], 'signal_threshold': [signal_threshold]})

>output_data(output_path, metadata, dff_traces, signal_features, save_as = 'csv', ROA_based = ROA_based, cell_assigned = cell_assigned, ROA_summary = ROA_summary)

## Expected outcomes

**Figure 6:**
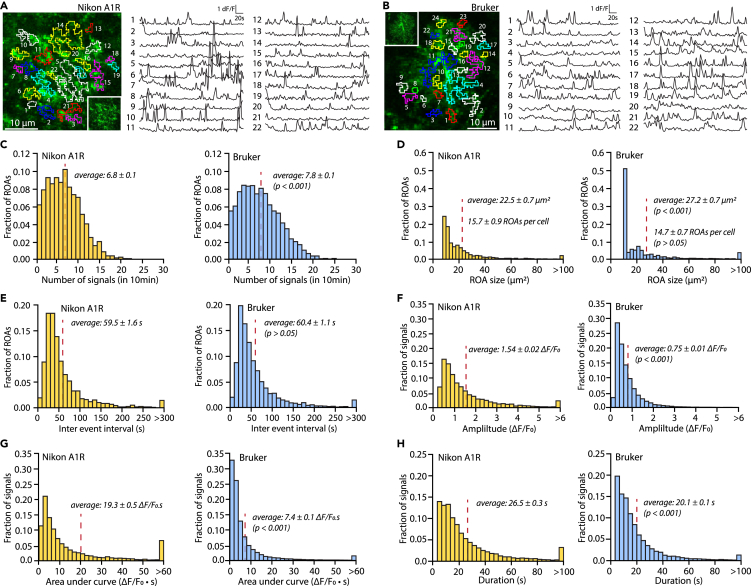
STARDUST benchmarking (A and B) Representative maps of ROAs overlaid on a maximum projection of an individual astrocyte, and fluorescence time series extracted from each ROA, for experiments conducted with a Nikon A1R (A) and a Bruker Ultima 2pPlus (B). Imaging conditions were the same otherwise (Nikon 25X, 1.10NA objective, laser power at 37.5 mW, recording rate 1 Hz, recording depth of 40 μm). Average signal to noise ratio of all slices collected from two microscopes are 3 and 2.5 respectively. Insets in (A) and (B) show the GCaMP6f fluorescence of the astrocyte under study (maximal projection). (C–E) Histograms illustrating the number of signals (C), ROA size (D), and inter-event interval distributions (E) across ROAs in 10 min recordings acquired with a Nikon A1R and Bruker 2-PLSM. Permutation tests were used, *n* = 3 slices from 2 mice. (F–H) Histograms illustrating the amplitude (F), area under the curve (D), and duration distributions (F) of signals detected in 10 min recordings acquired with a Nikon A1R and Bruker 2-PLSM. Permutation tests were used, *n* = 3 slices from 2 mice.

**Figure 7:**
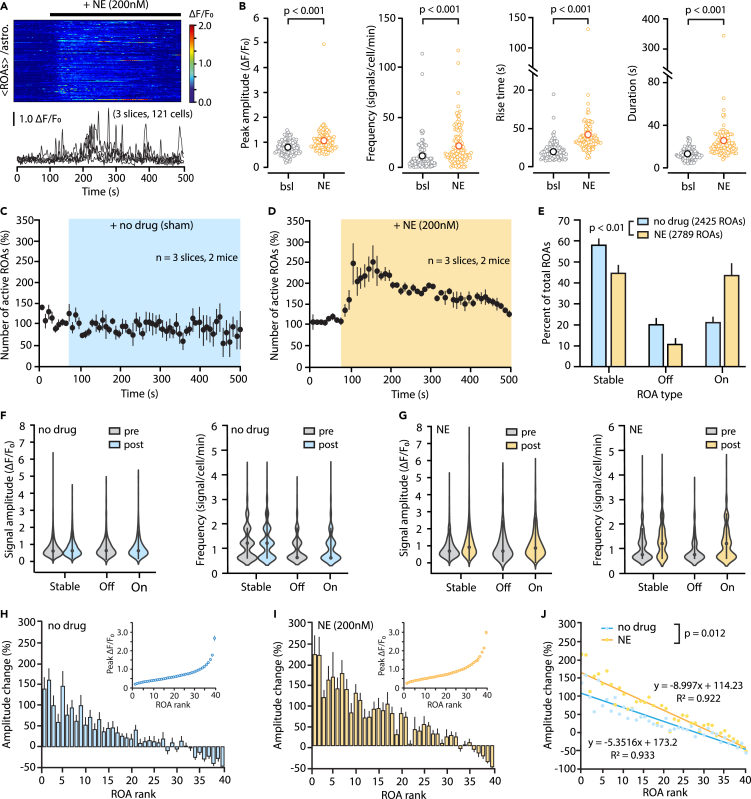
ROA-based analysis of calcium responses to norepinephrine with STARDUST (A) Kymograph (individual rows show averaged ROA fluorescence per astrocyte) and 5 representative ΔF/F_0_ traces (± SEM) of spontaneous astrocyte Ca^2+^ activity in response to the bath application of a ‘subthreshold’ concentration of NE (200 nM, i.e., causing no cell-wide response). (B) Plots showing the effect of 200 nM NE on the amplitude, frequency and kinetics of Ca^2+^ signals compared to the baseline epoch (bsl). Data are shown as averages of all ROAs per cell. Permutation tests were used. (C and D) Time courses of the effect of a no-drug sham application (C) and 200 nM NE bath application (D) on the number of active ROAs (10 s bins) normalized to baseline. Data shown as mean ±SEM. (E) Proportions of “Stable”, “On”, and “Off” ROAs for the experiments shown in (C) and (D) (no-drug vs. NE: *p* = 0.0019, Chi-square test). Data shown as mean ±SEM. (F and G) Signal amplitude and frequency per ROA type in no-drug (F) and NE (G) conditions. Effect of ‘no-drug’ on amplitudes across ROA types: *p* > 0.05, linear mixed effect model. Effect of ‘no-drug’ on frequency: Stable-pre vs. Off *p* < 0.001, vs. On *p* < 0.001, Stable-post vs. Off *p* < 0.001, vs. On *p* < 0.001. Effect of NE on amplitude: Stable-pre vs. post *p* < 0.001, vs. Off *p* < 0.001, vs. On *p* < 0.001. Effect of NE on frequency across ROA types: *p* < 0.001, linear mixed effect model. (H and I) ROA-ranked based analysis of the effect no-drug (H) and NE (I). ROAs are ranked based on the peak signal amplitude detected in the corresponding time-series during the baseline epoch (inset, 40 bins). The percentage change in amplitude after drug application is then plotted as a function of ROA rank. (J) Linear regression analysis of the experiments shown in (H) and (I). NE exerts a differential effect across ROA ranks that is greater than that observed by ‘chance’ (NE vs. No-drug, *p* = 0.012, combined regression model). Data are obtained from 100 s of pre-drug condition (0–100 s) and 100 s of post-drug condition (200–300 s) from stable ROAs.

In Figures 6 and 7, we illustrate some of the data that STARDUST yields in a classic experimental paradigm. Specifically, acute hippocampal brain slices were obtained from adult mice (P100) in which astrocytes were transduced with AAV5-gfaABC1D::lck-GCaMP6f at P70. GCaMP6f activity in *stratum radiatum* astrocytes was recorded under 2-PLSM. Figure 6 serves as a benchmark for STARDUST analysis output from data obtained under different recording conditions: Nikon A1RHD 25 MP microscope (920 nm excitation, Nikon 25X, 1.1 NA, 1.6x optical zoom, recorded at 40 μm depth) and Bruker Ultima 2pPlus (920 nm excitation, Nikon 25X, 1.1 NA, 1.6x optical zoom, record at 40 μm depth). As illustrated here, setup conditions could heavily affect data structure. We suggest that users be consistent in the setup used within a given dataset. Figure 7 provides example data on astrocyte calcium activity in response to the perfusion of low concentrations of norepinephrine (Tocris Biosciences, NE, 200 nM). Panels A-B show a classical cell-assigned ROA analysis of ΔF/F_0_ and signal properties across ROAs in response to NE with pairwise comparisons. Panels C-H provide examples of ROA-type and ROA-rank based analyses for the experiments shown in A-B.

## Limitations

The current version of STARDUST uses multiple software and platforms, which might prove strenuous for some users or require extra familiarization time. Authors will consider building an all-in-one user-friendly interface in future updates.

## Troubleshooting

### Problem 1

In ROA map acquisition (step 15), large, and connected ROAs arise.

### Potential solution

Reset filter criteria to have a smaller area range in step 12b. We encourage users to benchmark the output from a series of area ranges as shown in Figure 4 to find the optimal filter criteria. For example, the area ranges that give high signal to noise ratio and large number of ROAs could be good candidate values.

In addition, users can select a subset of frames to generate ROA maps in step 14 instead of the entire t-stack. This is especially helpful when a long recording was acquired (>15 min). We suggest keeping this criterion the same across experiments collected under the same conditions.

### Problem 2

When reading in the cell mask into Jupyter Notebook (step 31), the number of cells does not match the cell number delineated in ImageJ.

### Potential solution

When delineating the cells in step 23, cell boundaries should not touch each other, and the closet points should be a few pixels apart. Adjust cell mask accordingly in ImageJ.

### Problem 3

**Figure 8:**
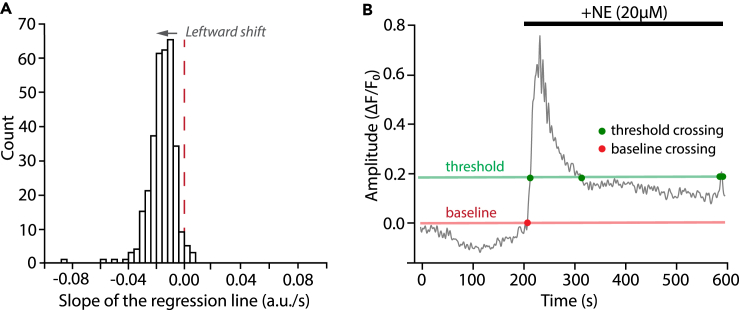
Troubleshooting illustration (A) Example of regression slope distribution from a recording with noticeable z-drift. The distribution is centered around −0.015. Also, note the lack of Gaussian symmetry (see troubleshooting 3). (B) Example of a time series in which NE application yielded a sustained signal, with no return to baseline within the recorded epoch, making baseline fluorescence determination and signal detection difficult (see troubleshooting 4).

Recording has a noticeable baseline intensity shift. This issue might become apparent at several steps in the STARDUST pipeline: when visually inspecting the recording, when plotting the z profile in ImageJ (step 3), or during the optional correction step in signal preprocessing section in Part 6 when the slope distribution of all traces is not centered around zero (Figure 8A).

### Potential solution

There are usually possible causes for a baseline intensity shift: 1) physical drifts in x, y, or z axis during the recording, 2) photo-bleaching, 3) biological change in baseline activities. The first two causes are technical and since both would significantly influence the signal detection, before attempting to compensate for the observed intensity shift, users should carefully examine the recording and decide whether to keep the recording for analysis. If users decide to keep the recording for analysis (in case of minor drifts or photo-bleaching), apply the optional correction step at signal preprocessing and proceed with the corrected traces for further analysis. The correction factor can be adjusted accordingly from 0 to 1, with 1 being the highest degree of correction. In cases where baseline fluorescence shift might be of biological origin, see troubleshooting 4 below.

### Problem 4

When applying pharmacology, the drug wash-in causes a large response that does not return to F_0_ before the recording ends, leading to failure in signal detection (since truncated signals are excluded in STARDUST default setting) (see Figure 8B).

### Potential solution

To rescue these truncated signals, users can specify an optional argument *include_incomplete* as True in the ***iterative_baseline*** function (step 34) using the code below. Note that only some of the signal stats, including amplitude, rise time, and peak time, will be outputted in the signal analysis summary. Read more about the function and its arguments in the help documentation by executing help(iterative_baseline) in a code block.

>dff_traces, baselines, thresholds, signal_frames, signal_boundaries, signal_threshold = iterative_baseline(corrected_traces, include_incomplete = True)

### Problem 5

A large percentage of ROAs are denoted as “inactive” in the output.

### Potential solution

In our conditions, the percent of ‘inactive” ROAs is usually 3%–10%. First, make sure the recording is stable and does not have major drifts on x, y, or z axis. If the analyzed recording is stable, a large proportion of “inactive” ROAs might denote 1) suboptimal voxel detection parameters in AQuA, 2) signal threshold being too high, 3) excessive time series with truncated signals (i.e., signals with no initiation or termination points), or 4) significant drifts in the baseline fluorescence, interfering with signal detection.

To identify the cause on a case-by-case basis, visually inspect the trace from these ROAs by using the ***inspect_trace*** function, with a list of ROA IDs, by running the code provided in the Jupiter CheckPoints.

>inactive_ROAs = df_ROA_cell[df_ROA_cell['ROA_type'] == 'inactive']['ROA_ID'].to_list()

>inspect_trace(inactive_ROAs, dff_traces, baselines, thresholds, drug_frame)

In case (1) where voxel detection parameters are too generous and thus the ROA map included many ROAs without true signals, increase the scaling factor in Part 2 step 10. In case (2) where the signal threshold is too high, rerun the code in the Baseline fluorescence determination and signal detection in steps 34–35, and use a lower threshold (for instance, 2SD instead of 3SD) when prompted. In case (3) where the presence of truncated signals is the major cause, refer to troubleshooting 4. Lastly, if the baseline fluorescence shows a noticeable drift (case (4)), consider applying a signal correction at the signal preprocessing step or other smoothing method such as sliding window for F_0_ determination (not currently supported by the pipeline).

## Resource availability

### Lead contact

Further information and requests for resources should be directed to and will be fulfilled by the lead contact, Thomas Papouin (thomas.papouin@wustl.edu).

### Technical contact

Questions about the technical specifics of performing the protocol should be directed to and will be answered by the technical contacts, Yifan Wu (yifan.wu@wustl.edu) and Yanchao Dai (d.miko@wustl.edu).

### Materials availability

This study did not generate new unique reagents.

### Data and code availability

All original code has been deposited at https://github.com/papouinlab/STARDUST. Example data has been deposited to Zenodo https://zenodo.org/doi/10.5281/zenodo.13126733.

## Acknowledgments

We would like to thank Dr. Peter Bayguinov from the Washington University Center for Cellular Imaging (WUCCI) for his help with 2-photon calcium imaging. We would also like to thank Manning Zhang for her help with executing motion correction. T.P. was supported by the National Institutes of Health (1R01MH127163-01), the DoD (W911NF-21-1-0312), the Brain & Behavior Research Foundation (NARSAD Young Investigator Award 28616), the Whitehall Foundation (2020-08-35), and the McDonnell Center for Cellular and Molecular Neurobiology Award (22-3930-26275U). The astrocyte 2-PLSM recordings were performed at the Washington University Center for Cellular Imaging (WUCCI) supported by Washington University School of Medicine, the Children’s Discovery Institute of Washington University and St. Louis Children’s Hospital (CDI-CORE-2015-505 and CDI-CORE-2019-813), and The Foundation for Barnes-Jewish Hospital (3770 and 4642). The graphical abstract and Figure 2 were created with BioRender.com.

## Author contributions

Y.W. led the project, developed the pipeline, analyzed data, wrote the first manuscript draft, made figures, and performed revisions. Y.D. developed the interactive versions of the pipeline, revised the code, made figures, edited the manuscript, and performed revisions. K.B.L. contributed example data and feedback. T.E.H. provided feedback on the pipeline. T.P. supervised the project, edited the manuscript, made figures, and assisted with revisions.

## Declaration of interests

The authors declare no competing interests.

## References

[1] Semyanov A., Henneberger C., Agarwal A. (2020). Making sense of astrocytic calcium signals - from acquisition to interpretation. Nat. Rev. Neurosci..

[2] Shigetomi E., Patel S., Khakh B.S. (2016). Probing the Complexities of Astrocyte Calcium Signaling. Trends Cell Biol..

[3] Rusakov D.A. (2015). Disentangling calcium-driven astrocyte physiology. Nat. Rev. Neurosci..

[4] Srinivasan R., Huang B.S., Venugopal S., Johnston A.D., Chai H., Zeng H., Golshani P., Khakh B.S. (2015). Ca(2+) signaling in astrocytes from Ip3r2(-/-) mice in brain slices and during startle responses in vivo. Nat. Neurosci..

[5] Arizono M., Inavalli V.V.G.K., Panatier A., Pfeiffer T., Angibaud J., Levet F., Ter Veer M.J.T., Stobart J., Bellocchio L., Mikoshiba K. (2020). Structural basis of astrocytic Ca2+ signals at tripartite synapses. Nat. Commun..

[6] Agarwal A., Wu P.-H., Hughes E.G., Fukaya M., Tischfield M.A., Langseth A.J., Wirtz D., Bergles D.E. (2017). Transient Opening of the Mitochondrial Permeability Transition Pore Induces Microdomain Calcium Transients in Astrocyte Processes. Neuron.

[7] Bindocci E., Savtchouk I., Liaudet N., Becker D., Carriero G., Volterra A. (2017). Three-dimensional Ca2+ imaging advances understanding of astrocyte biology. Science.

[8] Salmon C.K., Syed T.A., Kacerovsky J.B., Alivodej N., Schober A.L., Sloan T.F.W., Pratte M.T., Rosen M.P., Green M., Chirgwin-Dasgupta A. (2023). Organizing principles of astrocytic nanoarchitecture in the mouse cerebral cortex. Curr. Biol..

[9] Wang Y., DelRosso N.V., Vaidyanathan T.V., Cahill M.K., Reitman M.E., Pittolo S., Mi X., Yu G., Poskanzer K.E. (2019). Accurate quantification of astrocyte and neurotransmitter fluorescence dynamics for single-cell and population-level physiology. Nat. Neurosci..

[10] Bjørnstad D.M., Åbjørsbråten K.S., Hennestad E., Cunen C., Hermansen G.H., Bojarskaite L., Pettersen K.H., Vervaeke K., Enger R. (2021). Begonia—A Two-Photon Imaging Analysis Pipeline for Astrocytic Ca2+ Signals. Front. Cell. Neurosci..

[11] Dzyubenko E., Prazuch W., Pillath-Eilers M., Polanska J., Hermann D.M. (2021). Analysing Intercellular Communication in Astrocytic Networks Using “Astral.”. Front. Cell. Neurosci..

[12] Kluyver T., Ragan-Kelley B., PéRez F., Granger B., Bussonnier M., Frederic J., Kelley K., Hamrick J., Grout J., Corlay S., Loizides F., Schmidt B. (2016). Positioning and Power in Academic Publishing: Players, Agents and Agendas.

[13] Virtanen P., Gommers R., Oliphant T.E., Haberland M., Reddy T., Cournapeau D., Burovski E., Peterson P., Weckesser W., Bright J. (2020). SciPy 1.0: fundamental algorithms for scientific computing in Python. Nat. Methods.

[14] Harris C.R., Millman K.J., van der Walt S.J., Gommers R., Virtanen P., Cournapeau D., Wieser E., Taylor J., Berg S., Smith N.J. (2020). Array programming with NumPy. Nature.

[15] McKinney W. (2010). Data structures for statistical computing in Python. Proc. 9th Python Sci. Conf..

[16] Hunter J.D. (2007). Matplotlib: A 2D Graphics Environment. Comput. Sci. Eng..

[17] Waskom M. (2021). seaborn: statistical data visualization. J. Open Source Softw..

[18] Murray A., van Kemenade H., wiredfool, Clark J.A., Karpinsky A., Baranovič O., Gohlke C., Dufresne J., Yay295, Brett M. (2024).

[19] Schindelin J., Arganda-Carreras I., Frise E., Kaynig V., Longair M., Pietzsch T., Preibisch S., Rueden C., Saalfeld S., Schmid B. (2012). Fiji: an open-source platform for biological-image analysis. Nat. Methods.

[20] Mi X., Chen A.B.-Y., Duarte D., Carey E., Taylor C.R., Braaker P.N., Bright M., Almeida R.G., Lim J.-X., Ruetten V.M.S. (2024). Fast, Accurate, and Versatile Data Analysis Platform for the Quantification of Molecular Spatiotemporal Signals. bioRxiv.

[21] Butterworth S. (1930). On the Theory of Filter Amplifier. Experimental Wireless and the Wireless Engineer.

